# The KEAP1–NRF2 System as a Molecular Target of Cancer Treatment

**DOI:** 10.3390/cancers13010046

**Published:** 2020-12-26

**Authors:** Keiko Taguchi, Masayuki Yamamoto

**Affiliations:** 1 Department of Medical Biochemistry, Graduate School of Medicine, Tohoku University, Sendai 980-8575, Japan; keiko@med.tohoku.ac.jp; 2 Department of Medical Biochemistry, Tohoku Medical Megabank Organization, Tohoku University, Sendai 980-8573, Japan; 3 Advanced Research Center for Innovations in Next-Generation Medicine (INGEM), Tohoku University, Sendai 980-8573, Japan

**Keywords:** NRF2, KEAP1, adaptive response, NRF2-addicted cancers

## Abstract

**Simple Summary:**

Nuclear factor erythroid-derived 2-like 2 (encoded by the *Nfe2l2* gene; NRF2) is a transcription factor that regulates a variety of cytoprotective genes, including antioxidant enzymes, detoxification enzymes, inflammation-related proteins, drug transporters and metabolic enzymes. NRF2 is regulated by unique molecular mechanisms that stem from Kelch-like ECH-associated protein 1 (KEAP1) in response to oxidative and electrophilic stresses. It has been shown that disturbance or perturbation of the NRF2 activation causes and/or exacerbates many kinds of diseases. On the contrary, aberrant activations of NRF2 also provoke intriguing pathologic features, especially in cancers. Cancer cells with high NRF2 activity have been referred to as NRF2-addicted cancers, which are frequently found in lung cancers. In this review, we summarize the current accomplishments of the KEAP1–NRF2 pathway analyses in special reference to the therapeutic target of cancer therapy. The concept of synthetic lethality provides a new therapeutic approach for NRF2-addicted cancers.

**Abstract:**

The Kelch-like ECH-associated protein 1 (KEAP1)—Nuclear factor erythroid-derived 2-like 2 (encoded by the *Nfe2l2* gene; NRF2) system attracts extensive interest from scientists in basic and clinical cancer research fields, as NRF2 exhibits activity as both an oncogene and tumor suppressor, depending on the context. Especially unique and malignant, NRF2-addicted cancers exhibit high levels of NRF2 expression. Somatic mutations identified in the *NRF2* or *KEAP1* genes of NRF2-addicted cancers cause the stabilization and accumulation of NRF2. NRF2-addicted cancers hijack the intrinsic roles that NRF2 plays in cytoprotection, including antioxidative and anti-electrophilic responses, as well as metabolic reprogramming, and acquire a marked advantage to survive under severe and limited microenvironments. Therefore, NRF2 inhibitors are expected to have therapeutic effects in patients with NRF2-addicted cancers. In contrast, NRF2 activation in host immune cells exerts significant suppression of cancer cell growth, indicating that NRF2 inducers also have the potential to be therapeutics for cancers. Thus, the KEAP1–NRF2 system makes a broad range of contributions to both cancer development and suppression. These observations thus demonstrate that both NRF2 inhibitors and inducers are useful for the treatment of cancers with high NRF2 activity.

## 1. Molecular Mechanisms Underlying NRF2 Activation

Our body is constantly exposed to a variety of chemical stresses from external environments, including natural ultraviolet radiation, air pollutants from urban industries and water pollutants from micro/nanoplastic particles [1]. Our personal environments, including lifestyle and food, drinking water, tobacco, alcohol and drug consumption, increase the risk of taking in toxic chemicals that produce oxidative and electrophilic stresses and damage macromolecules, such as nucleic acids, proteins and lipids [2]. However, our body is equipped with the ability to eliminate these toxic xenobiotics and adapt to the environment, which we refer to as the adaptive response or the environmental stress response [3,4]. Environmental stresses often perturb intracellular conditions that challenge our body’s homeostasis at the levels of genes, proteins and metabolites, but the adaptive response protects our body from these challenges. In fact, the adaptive response is essential for our survival in modern society. To attain the adaptation ability to overcome the toxicity of environmental xenobiotics and recover a steady state, our body needs to sense the stresses and convert them into intra- and intercellular signals.

**Figure 1:**
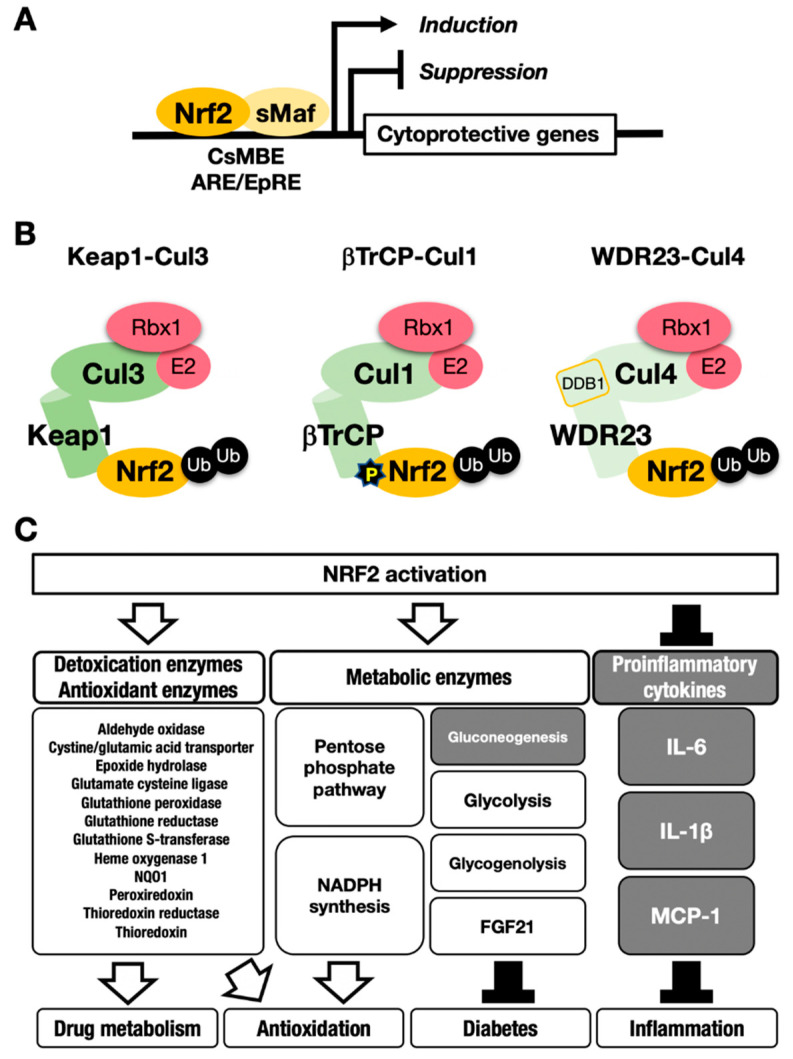
The NRF2-sMaf heterodimer regulates cytoprotective gene expression through the CNC-sMaf-binding element (CsMBE) motif. (**A**) NRF2 forms a heterodimer with sMaf and binds to the CsMBE motif in the nucleus, which is classically referred to as an antioxidant responsive element (ARE)/electrophile responsive element (EpRE) motif. NRF2 positively regulates genes encoding detoxicating enzymes and antioxidative enzymes and negatively regulates genes coding for proinflammatory factors. Ub, ubiquitin. P, phosphorylation. (**B**) The combination of an E3 ligase and the adaptor for the degradation of NRF2 through ubiquitination: KEAP1–Cul3, βTrCP–Cul1 and WDR23-Cul4 complexes. (**C**) NRF2 target genes responsible for the drug metabolism, antioxidation, antidiabetes and anti-inflammation. White arrows indicate induction; black bars indicate suppression.

Nuclear factor erythroid-derived 2-like 2 (encoded by the *Nfe2l2* gene; NRF2) is a transcription factor that controls the environmental stress response by changing gene expression profiles [5]. NRF2 regulates a subset of target genes that mainly encode cytoprotective enzymes/proteins critical to the antioxidative response and detoxication [6] (Figure 1A). NRF2 is activated when cells are exposed to oxidative stresses or toxic chemicals (many of which are electrophilic) [7,8]. This activation of NRF2 is fine-tuned by Kelch-like ECH-associated protein (KEAP11), an adaptor for Cullin 3 (Cul3)-based ubiquitin E3 ligase [9] (Figure 1B, left). KEAP1 binds NRF2 and promotes the ubiquitination of NRF2. Under steady-state conditions, ubiquitinated NRF2 is rapidly degraded by the 26S proteasome. NRF2 has a very short half-life, which is less than 20 min [9,10]. Therefore, NRF2 does not exist abundantly under basal conditions, and available lines of evidence support the contention that NRF2 exists at a relatively low level in most organs or tissues. Consistent with this assertion, juvenile *NRF2*-knockout mice and rats do not show apparent external phenotypes, except for white teeth [11,12,13].

Ubiquitin–proteasome system-based protein degradation is evident in a number of important regulatory systems [14]. For instance, an inhibitor of the nuclear factor-κB (IκB)-NFκB system, hypoxia-inducible factor (HIF) system and estrogen receptor-α (ERα) system are known to function in response to ubiquitination by specific ubiquitin E3 ligases (e.g., Cul1, Cul2 or Cul4B) and corresponding adaptors. Of the ubiquitin–proteasome-based regulatory systems, the KEAP1–NRF2 system is unique in that it can sense oxidative and electrophilic stresses through the reactive cysteine residues within KEAP1 and mediates the expression of cytoprotective enzyme genes through NRF2 activity. In this system, KEAP1 acts as a sensor for stress, and NRF2 acts as a transcription factor that activates cytoprotective gene expression. Once our bodies are exposed to reactive oxygen species (ROS) or electrophilic toxicants, reactive cysteine residues in KEAP1 are covalently modified by ROS or electrophiles, which stops NRF2 ubiquitination. The activity of modified KEAP1 is weakened, resulting in newly transcribed NRF2 escaping ubiquitination and proteasomal degradation.

This process leads to NRF2 accumulation at the protein level, which induces robust transactivation of cytoprotective genes [7,8] (Figure 1C). NRF2 upregulates the expression of genes encoding detoxicating enzymes and antioxidative enzymes such as NAD(P)H:quinone oxidoreductase 1 (*Nqo1*) and heme oxygenase-1 (*Ho-1*, encoded by the *Hmox1* gene).

In contrast, NRF2 downregulates genes encoding proinflammatory factors such as interleukin-6 (*IL6*) and interleukin 1β (*IL1β*) [15]. NRF2 acts as a transcription factor by forming a heterodimer with a small Maf protein (sMaf), including MafF, MafG or MafK, and by binding to the CNC-sMaf-binding element (CsMBE) (Figure 1A), which is classically defined as an antioxidant responsive element (ARE) or electrophile responsive element (EpRE) [6]. The rapid degradation of NRF2 under normal conditions and quick stabilization upon exposure to stresses allows quick and urgent responses to ROS and/or toxic chemicals (often electrophiles). These are critical features utilized commonly in the environmental stress response systems. Typical examples of such environmental stress response systems are the systems regulated by transcription factors NRF2 and HIF. The activation is evoked by the derepression that halts the repression by constant proteasomal degradation of effector transcription factors.

As NRF2 is regulated at the posttranscriptional level and as NRF2 is an unstable protein, NRF2 activation has been monitored by several indirect approaches, such as monitoring the expression of NRF2 target genes or knocking in fluorescent proteins. Of the NRF2 target genes, Nqo1 encodes a representative marker enzyme that has been exploited for the evaluation of NRF2 activity [11]. In NRF2-induction experiments utilizing cell culture systems, *Nqo1* mRNA is usually highly upregulated approximately 12 h and longer after exposure to electrophilic NRF2-inducers [16]. The other representative target genes of NRF2 are glutamate-cysteine ligase catalytic or modifier subunits (*Gclc or Gclm*) for glutathione (GSH) synthesis [14] and show a similar response profile as *Nqo1* mRNA. In contrast, while the *Ho-1* gene is also an important NRF2 target, upon induction, *Ho-1* gene expression peaks approximately 3 h after a challenge by electrophilic inducers. It has been reported that the *Ho-1* gene is under complex regulation, which may elicit the early induction peak [16]. In fact, the upregulation of *Ho-1* mRNA levels was not observed in liver-specific *KEAP1*-knockout mice, even though *Nqo1* mRNA levels were upregulated and reflected the NRF2 activation level [17].

## 2. Factors Critical for the Rapid Degradation of NRF2

### 2.1. KEAP1

After the discovery of NRF2 [18,19], its Neh2 domain was soon identified as essential for high-level NRF2 activity in a chicken erythroid cell line [20]. As an approach to delineate the domain function, a yeast two-hybrid screen was conducted using the Neh2 domain as bait, and we identified a new molecule, KEAP1 [20]. KEAP1 is similar to the human protein KIAA0132, which has an unknown function. Molecular dissection of KEAP1 revealed that the protein contains a bric-a-brac, tramtrack, broad complex (BTB) domain, an intervening region (IVR) and 6 Kelch domains that are also referred to as double-glycine repeat (DGR) domains [20].

**Figure 2:**
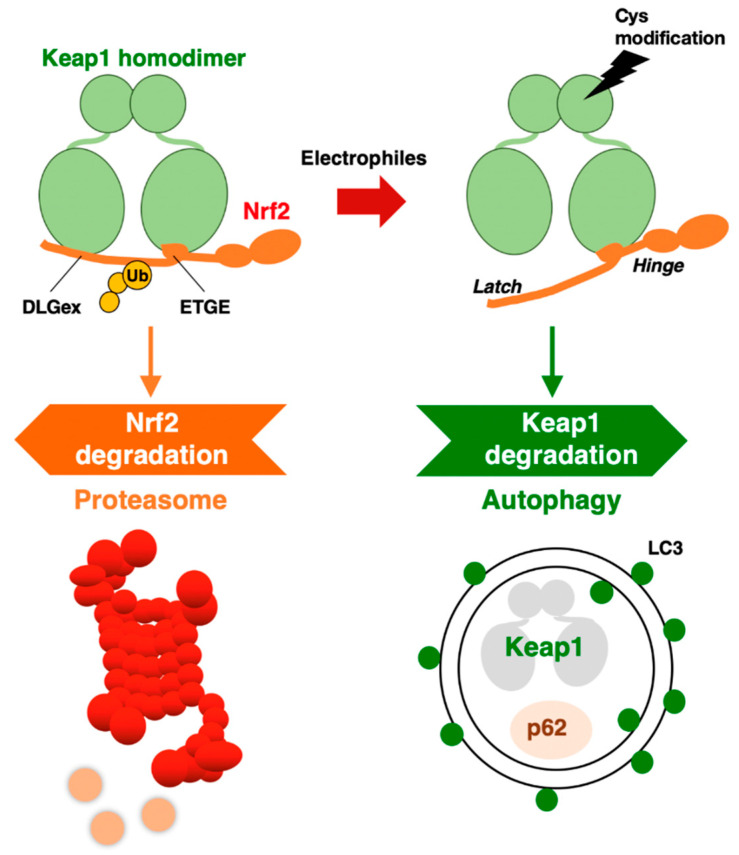
KEAP1–NRF2 system: protein regulation by both proteasome and autophagy.

Through the DGR domain, a KEAP1 homodimer binds to DLGex and ETGE motifs in the Neh2 domain of NRF2 [21,22,23] (Figure 2). Interestingly, the ETGE motif binds to KEAP1 with approximately 100-fold higher affinity than the DLGex motif (Figure 2). This difference in the binding affinity of KEAP1 and DLGex as well as ETGE motifs led to the proposal of the hinge–latch model [22]. Deletion of the DGR domain in KEAP1 causes constitutive activation of NRF2 due to the cessation of the ubiquitination and proteasomal degradation of NRF2 [20]. The KEAP1-mediated repression of NRF2 activity is critical for our bodies, as indicated by the targeted deletion of KEAP1 in mice resulting in lethal hyperkeratosis in the upper digestive tract [24,25]. Simultaneous NRF2 deletion attenuates the phenotype, unequivocally demonstrating that aberrant NRF2 activation provokes hyper-keratinization in *KEAP1*-knockout mice [24]. Experiments using several *KEAP1* gene-modified mouse strains have shown that the KEAP1-mediated regulation of NRF2 activity is fine-tuned according to the KEAP1 expression level [17].

The KEAP1 homodimer binds to NRF2 through the DLGex and ETGE motifs in the Neh2 domain. The binding affinity of the ETGE motif for KEAP1 is approximately 100-fold stronger than that of DLGex. The KEAP1–Cul3 complex ubiquitinates NRF2, which is then degraded by the 26S proteasome. When electrophiles modify reactive cysteine residues in KEAP1, the DLGex motif with weaker affinity releases NRF2, but the ETGE motif maintains binding (hinge–latch model). Electrophilic modification of KEAP1 cysteine residues leads to KEAP1 degradation by autophagy. In the impaired autophagy situation, KEAP1 protein accumulates with p62.

### 2.2. βTrCP

Phosphatidylinositol-3 kinase (PI3K) is activated by receptor tyrosine kinases, and it phosphorylates phosphatidylinositol-4,5-bisphosphate (PIP_2_) to generate phosphatidylinositol-3,4,5-triphosphate (PIP_3_). In contrast, Pten (phosphatase and tensin homolog deleted on chromosome 10) dephosphorylates PIP_3_ to PIP_2_ and thus counteracts the action of PI3K [26]. v-akt murine thymoma viral oncogene homolog (protein kinase B; Akt) is activated via phosphorylation. The phosphorylation of Akt at Thr308 by PDK1 (3-phosphoinositide-dependent kinase-1) primes the kinase to be phosphorylated further at Ser473 by mammalian target of rapamycin complex 2 (mTORC2). Activated Akt then phosphorylates a number of physiologically important substrates that promote cell survival, migration, cell cycle progression, and metabolism. For instance, glycogen synthase kinase 3β (GSK3β) is an important substrate of Akt. Activated Akt phosphorylates GSK3β and inactivates GSK3β.

It has been shown that NRF2 is phosphorylated by GSK3β at Ser344 and Ser347 in humans (Ser335 and Ser338 in mice). These residues are located in the Neh6 domain of NRF2 [27,28]. β-transducin repeat-containing E3 ubiquitin–protein ligase (βTrCP)–Cullin1 (Cul1) complex has been identified as an alternative pathway for NRF2 degradation, in addition to the KEAP1–Cul3 pathway (Figure 1B, middle) [25]. The βTrCP–Cul1 complex ubiquitinates GSK3β-phosphorylated NRF2 in the nucleus, and ubiquitinated NRF2 is degraded by the 26S proteasome. However, in *Pten*-deleted livers of juvenile mice, GSK3β inactivation elicited by Akt activation disrupts the accumulation of substantial amounts of NRF2 in the nucleus [25], indicating that inactivation of the βTrCP–Cul1 pathway alone is not strong enough to cause NRF2 accumulation. In contrast, simultaneous Pten deletion and KEAP1 deletion in the liver synergistically lead to the accumulation of NRF2 compared with the effect of KEAP1-deletion alone or Pten-deletion alone. These results support the notion that the KEAP1–Cul3 pathway in the cytoplasm is the predominant pathway of NRF2 degradation, whereas the βTrCP–Cul1 pathway in the nucleus serves as a supplemental regulation system for the degradation of NRF2. Alternatively, the βTrCP–Cul1 pathway may contribute to certain organs or tissues that are dependent on it.

### 2.3. WDR23

Mammalian NRF2 is an ortholog of *Caenorhabditis elegans* SKN-1 (Skinhead-1) [29]. *C. elegans* lacks the KEAP1 ortholog. SKN-1 is ubiquitinated by WDR23 (WD40-repeat protein-23), which forms a complex with DDB1 (damaged DNA-binding protein 1) and Cul4, and ubiquitinated SKN-1 is degraded by the proteasome [30]. It has been shown that WDR23 directly regulates NRF2 degradation in human cells [31] (Figure 1B, right), and the suppression of WDR23 leads to the upregulated expression of cytoprotective genes in mammalian cells, as is the case for *C. elegans* [32]. Human WDR23 isoforms 1 and 2 are mainly localized in the cytoplasm and nucleus, respectively. WDR23 isoform 1 may regulate the cytosolic NRF2 level in cooperation with KEAP1.

WDR23 binds to the DIDLID element (amino acids 17–32; MDLIDILWRQDIDLG) [33] that localizes in the proximity of the DLGex motif (amino acids 17–51) and upstream of the ETGE motif (amino acids 79–82) within the Neh2 domain of NRF2 [31]. The DIDLID element is conserved in *C. elegans* SKN-1 and in mice, rats and humans NRF2 [33]. In the Neh2 domain of NRF2, which binds to WDR23 and KEAP1, the DIDLID, DLGex and ETGE motifs may contribute to the turnover of the NRF2 protein. Further detailed experiments are needed to show the importance of WDR23-Cul4 pathway contributions to the regulation of NRF2.

## 3. Autophagic Degradation of KEAP1

NRF2 protein is degraded through the ubiquitin–proteasome system. On the other hand, when we focus on the turnover of KEAP1, it has been shown that KEAP1 is also ubiquitinated in a Cul3-dependent manner, but the 26S proteasome system does not degrade KEAP1 [34]. Furthermore, when cells are exposed to an electrophilic NRF2-inducer, such as *tert*-butylhydroquinone (tBHQ), the KEAP1 protein is ubiquitinated, and its level is decreased [34]. Therefore, it was concluded that KEAP1 is degraded in a “proteasome-independent” manner. However, it took several years after the paper was published to clarify the molecular mechanism underlying KEAP1 protein turnover.

A clue for this question emerged from the study of p62 (the *Sqstm1* gene product). In autophagy-impaired livers, a massive accumulation of p62 is routinely observed concomitant with NRF2 activation. When we examined these livers, we accidentally found abundant KEAP1 protein accumulation [35], suggesting that KEAP1 is degraded through the autophagy pathway. As p62 is a scaffold or chaperone protein in autophagy [36], the most plausible scenario is that p62 binds to KEAP1 and brings KEAP1 to the autophagosome. We have demonstrated this process using several approaches. In the *p62*-knockout mouse liver, *KEAP1* mRNA levels are unchanged, but KEAP1 protein levels are increased, suggesting that KEAP1 is stable without p62 guidance to autophagosomes. p62 binds to KEAP1 through an STGE motif (amino acids 349–352 in mouse/351–354 in human) in the (KEAP1-interacting region (KIR), and phosphorylation of the STGE motif (i.e., pSTGE) increases the binding affinity as the pSTGE motif mimics the ETGE motif in NRF2 [37]. The binding of p62 to KEAP1 through the pSTGE motif results in the inactivation of the ubiquitin ligase activity of KEAP1, indicating that, in impaired autophagy, p62 accumulation activates NRF2 due to the disruption of KEAP1 binding to NRF2. In fact, starvation-induced autophagy accelerated the degradation of the KEAP1 protein [35].

These extensive observations support the notion that KEAP1 is selectively degraded through autophagy, but not the proteasome. The degradation of specific proteins through autophagosomes is referred to as selective autophagy. Thus, the KEAP1–NRF2 system is regulated by two protein degradation systems: the ubiquitin–proteasome system and the selective autophagy system (Figure 2).

An intriguing finding along this line is that while the normal half-life of KEAP1 degradation is 12.7 h, the half-life of its degradation is shortened to 3.4 h by tBHQ exposure [35]. In addition to tBHQ, other electrophilic NRF2 inducers that modify KEAP1 cysteine residues, such as diethyl maleate (DEM) and 1,2-naphthoquinone, have also been shown to accelerate KEAP1 degradation. Importantly, the proteasome inhibitor MG132 was reproducibly ineffective at the KEAP1 protein level [34,35], indicating that the 26S proteasome is not critical for KEAP1 degradation.

## 4. Additional Substrates of KEAP1

It has been reported that in addition to NRF2, KEAP1 ubiquitinates several other substrates. For instance, the following proteins have been reported as candidates: mitochondrial serine/threonine protein phosphatase PGAM family member 5 (PGAM5) [38], NFκB kinase inhibitor IKKβ (inhibitor of nuclear factor-kappa B kinase subunit β, referred to as IKBKB) [39], BRCA2 partner PALB2 (partner and localizer of BRCA2) [40], and replicative helicase subunit protein MCM3 (minichromosome maintenance complex component 3) [41]. These proteins are reported to contain ETGE or ETGE-like motifs, which are required for binding to KEAP1 [42]. However, the mechanism by which KEAP1 has an impact on the functions of these substrates and how KEAP1 exhibits structural affinities for them remain unclear. While the physiological significance of KEAP1 as an adaptor for NRF2 ubiquitination has been examined extensively, aspects of the aforementioned substrate proteins remain to be explored.

In this regard, it is interesting to note that p62 also has been shown to be a substrate of the KEAP1–Cul3 complex. p62 is a well-characterized protein that binds to KEAP1 in physiological and protein structure analyses. Although in a manner different from that of the aforementioned observations, it has been reported that p62 is ubiquitinated by KEAP1 at Lys420 in the ubiquitin-associated (UBA) domain [43], resulting in its degradation by autophagy [44]. Dimerization of p62 through the Phox1 and Bem1p (PB1) domain is required for ubiquitination. Nonetheless, the physiological contribution of the KEAP1-mediated ubiquitination of p62 remains to be clarified.

## 5. Monitoring NRF2 Activity for Clinical Applications

### 5.1. Importance of Biomarkers for Monitoring NRF2 Activity

Gene expression analysis of peripheral blood from individuals is emerging as an important approach for personalized healthcare and medicine. In fact, recent studies have demonstrated that single nucleotide polymorphisms (SNPs) in the human N*RF2* promoter are associated with higher risks for the development of acute lung injury [45], ulcerative colitis [46], nephritis [47], vascular stiffness with aging [48] and cancers [49,50]. SNPs in the *NRF2* promoter determine *NRF2* expression levels in individuals. Therefore, noninvasive monitoring of NRF2 activity will be important for the applications of NRF2-targeted drugs and/or for the assessment of the therapeutic effects of the drugs in individuals with different NRF2 expression levels. We expect that NRF2 activation status in patients and healthy people will be routinely evaluated in the near future.

**Figure 3:**
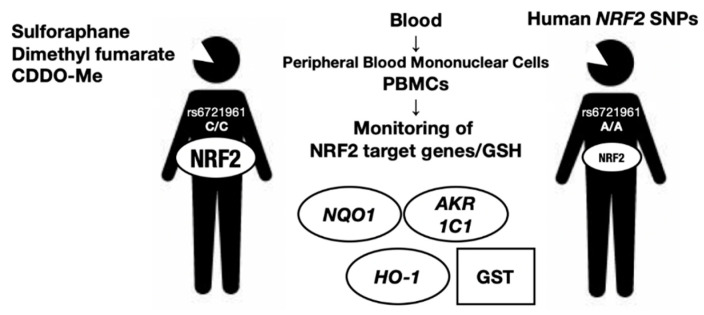
Monitoring of NRF2 activity in human blood samples. Human *NRF2* rSNP, rs6721961, is located within an ARE motif 617 bp upstream from the transcription start site of the *NRF2* gene. The minor A/A homozygote diminishes *NRF2* gene expression by 40% compared with the C/C homozygote or C/A heterozygote. In diseases including cancers, chronic obstructive pulmonary disease (COPD), autism and multiple sclerosis, NRF2 activity is a potential biomarker for NRF2-inducers including sulforaphane, dimethyl fumarate and bardoxolone methyl (CDDO-Me) that can be monitored by glutathione (GSH) and NRF2 target genes.

In this regard, measurement of the expression levels of NRF2 target genes may be one of the most convenient approaches for monitoring NRF2 activity and/or predicting individual risk for acquiring an NRF2-related disease. For example, *Aldo-keto reductase* (*AKR*) *1C1*, an NRF2 target gene, has been suggested to be a good marker for NRF2 activity in human peripheral blood cells [51]. We surmise that many other markers reflecting NRF2 activity in peripheral blood cells and in specific tissues or organs will be available in the near future. The ability to monitor the genetic and pharmacological activation of NRF2 by examining NRF2 target gene expression in peripheral blood samples may be realized soon. We believe that this advancement will benefit many aspects of personalized medicine and healthcare-related to NRF2 activity. However, monitoring NRF2 activity in vivo has not progressed to satisfactory levels to date. We succinctly describe some of the current examples of NRF2 activity monitoring using human cases (Figure 3).

NRF2 activity in peripheral blood mononuclear cells is able to be measured by means of GSH level of NRF2 target gene expression levels and acts as an appropriate biomarker. *NRF2* SNPs are also shown to affect the NRF2 activity levels in peripheral blood mononuclear cells.

### 5.2. Monitoring NRF2 Activity in Cancer Cases

Human SNPs in the *NRF2* locus can be genotyped routinely using genomic DNA extracted from whole blood samples. A regulatory SNP (rSNP), rs6721961, is located within an ARE motif 617 bp upstream from the transcription start site of the *NRF2* gene [49]. The minor A/A homozygote rSNP–617 diminishes *NRF2* gene expression by 40% compared with the C/C homozygote or C/A heterozygote because of the weakened binding affinity of NRF2 with the CsMBE motif. In tBHQ-treated lymphocytes, the *NQO1* mRNA expression level decreases stepwise according to the presence of the A allele, i.e., the A/A variant has the lowest expression level. Decreased expression of NRF2 in A/A homozygotes has been shown to be correlated with increased risk of acute lung injury [45] and lung cancer incidence, especially in patients who have ever smoked [49]. Therefore, an inspection of the *NRF2* rSNP seems to be useful for lung cancer prevention. Similarly, an rSNP is also found in the mouse *NRF2* locus [52].

Aberrant activation of NRF2 has been shown to provoke malignant growth and anticancer drug resistance in many types of cancers [53] (see Section 6). Salient examples for these NRF2-activated (or NRF2-addicted) malignant cancers [54] can be seen in non-small cell lung cancers [55]. In this regard, NRF2 and its target gene expression levels in human cancer biopsy samples served as biomarkers for the diagnosis of these cancers. AKRs, including the AKR1B and AKR1C1/2/3 isozymes, at both the mRNA and protein levels were shown to be biomarkers for the diagnosis of NRF2-activated cancers in the lung [56]. Although NRF2 can be used as a biomarker, AKRs are detected with higher sensitivity than NRF2. We surmise that the precise diagnosis of constitutive NRF2 activation in lung cancers will improve the selection of anticancer drugs.

Bardoxolone methyl (CDDO-Me, 2-cyano-3,12-dioxooleana-1,9-dien-28-oic acid methyl ester) is a synthetic triterpenoid that markedly induces NRF2 [57,58,59]. Currently, CDDO-Me is under the phase 3 clinical trial for diabetic kidney disease in Japan (Ayame study, ClinicalTrials.gov: NCT03550443). Intriguingly, CDDO-Me was first developed as an anticancer reagent. A phase I first-in-human trial of CDDO-Me enrolled patients with advanced solid tumors and lymphomas [60]. The plasma concentration of CDDO-Me for the patients treated with the first daily oral dose of 900 mg during a 28-day cycle peaked 4 h after administration on day 21 and then decreased very slowly within 48 h. CDDO-Me remained in the plasma for more than 24 h after dosing. This pharmacological NRF2 activation by CDDO-Me was assessed by the expression level of *NQO1* mRNA in human peripheral blood cells from the CDDO-Me-treated patients. This study clearly demonstrates that, after induction, NRF2 activity can be monitored by target gene expression in human blood.

In this regard, we also surmise that there are limitations of monitoring NRF2 activity in the circulatory system. For instance, NRF2 expression can be different on certain occasions between the circulatory system and pathogenic site of the disease.

### 5.3. Monitoring NRF2 Activity in Other Chronic Diseases

Chronic obstructive pulmonary disease (COPD) refers to collective chronic inflammatory lung diseases that cause obstructed airflow from the lungs. The mRNA and protein expression levels of *NRF2* and its target genes *HO-1* and *GCLC* are increased in peripheral blood mononuclear cells derived from mild-moderate COPD cases [61,62]. The severity of COPD is correlated with the expression levels of *NRF2* and its target genes. In a longitudinal observational study, the GSH levels in plasma and the expression levels of NRF2-related genes in peripheral blood mononuclear cells appeared to serve as indicative biomarkers for the progression of disease in COPD patients [61,62].

Autism spectrum disorder is among the most common neurodevelopmental disorders. It is interesting to note that a dietary phytochemical, sulforaphane [1-isothiocyanato-4-(methylsulfinyl)-butane], shows efficacy for the disorder. Sulforaphane is derived from the inactive precursor glucoraphanin, which is abundant in broccoli (*Brassica oleracea var. italica*) sprouts. Glucoraphanin is converted to sulforaphane by myrosinase, which is produced by the microflora of the gastrointestinal tract [63]. Sulforaphane has cytoprotective and anti-inflammatory activities dependent on NRF2 [64]. It has been reported that sulforaphane alleviates abnormal cognition and behavior in people with autism spectrum disorder [65]. In peripheral blood mononuclear cells from autism spectrum patients treated with orally delivered sulforaphane, the expression levels of the mRNAs for cytoprotective enzymes, including *NQO1*, *HO-1*, and *AKR1C1,* were increased; in contrast, the expression levels of proinflammatory cytokines, including *IL*6, *IL1β*, *COX2* and *TNFα*, were decreased [66]. With these blood biomarkers, it seems possible to monitor pharmacodynamic responses to sulforaphane in both healthy humans and those with autism.

Relapsing-remitting multiple sclerosis is an autoimmune inflammatory disease of the central nervous system. Dimethyl fumarate (DMF, Tecfidera^®^) is an oral formulation of fumaric acid esters that has been approved for the treatment of relapsing-remitting multiple sclerosis with relapses, disability progression, and inflammatory tissue lesions. DMF also leads to enhanced immunomodulatory and antioxidant actions, leading to neuroprotection [67]. DMF reduces cytokine production and immune cell migration by activating NRF2 [68]. Patients with higher *NQO1* levels in peripheral blood samples 4–6 weeks after DMF therapy initiation are likely to achieve disease-free status within one year, suggesting that DMF-induced NRF2 activation may be the mechanism of action of therapeutic DMF in this disease. NRF2 activity is a potential biomarker for DMF treatment that can be monitored by means of *NQO1* mRNA expression in peripheral blood samples. In this regard, we surmise that the *NRF2* rSNP described herein may impact the pharmacological effect of NRF2-inducers in multiple sclerosis cases.

## 6. Roles of NRF2 in Cancer Progression

### 6.1. NRF2-Addicted Cancers

There are a tremendous number of reports that show somatic mutations in the *KEAP1* and *NRF2* genes in cancers that originated in various tissues, and their frequencies are especially high in non-small cell lung carcinomas [55,69]. The DLGex and ETGE motifs of NRF2 are two hot spots of somatic mutations in the *NRF2* gene, and this observation must have some mechanistic implications [54]. Somatic mutations in the *KEAP1* or *NRF2* genes cause constitutive NRF2 activation through disruption of the protein–protein interaction (PPI) between KEAP1 and NRF2. In addition to these mutations, oncometabolites [70,71], exon skipping [72], and promoter methylation [73] lead to constitutive NRF2 activation in cancers [14,74]. Furthermore, *NRF2* gene expression is transcriptionally regulated by oncogenes, such as *K-Ras* and *c-Myc* [75]. Cancer cells with these somatic mutations and the resulting high levels of NRF2 activity are referred to as NRF2-activated or NRF2-addicted cancers, which retain malignant growth with increased proliferation ability and potentiated resistance to chemo- and radiotherapy [76]. To treat these NRF2-addicted cancers, NRF2 inhibitors are needed that exert therapeutic effects [54].

When designing effective treatments for NRF2-addicted cancers, various synergistic and additive interactions of NRF2 with other regulatory factors need to be considered. For instance, PTEN, a tumor suppressor that negatively regulates the PI3K-Akt pathway, upregulates NRF2 activity [77]. Loss of PTEN function in cancers activates Akt phosphorylation of downstream factors, including GSK3β. In 80% of human *PTEN*-deficient endometrioid tumors, NRF2 is overexpressed in accordance with HO-1 upregulation [77]. Consistently, experiments with cell cultures and mouse models revealed that loss of PTEN activates NRF2 [77,78].

**Figure 4:**
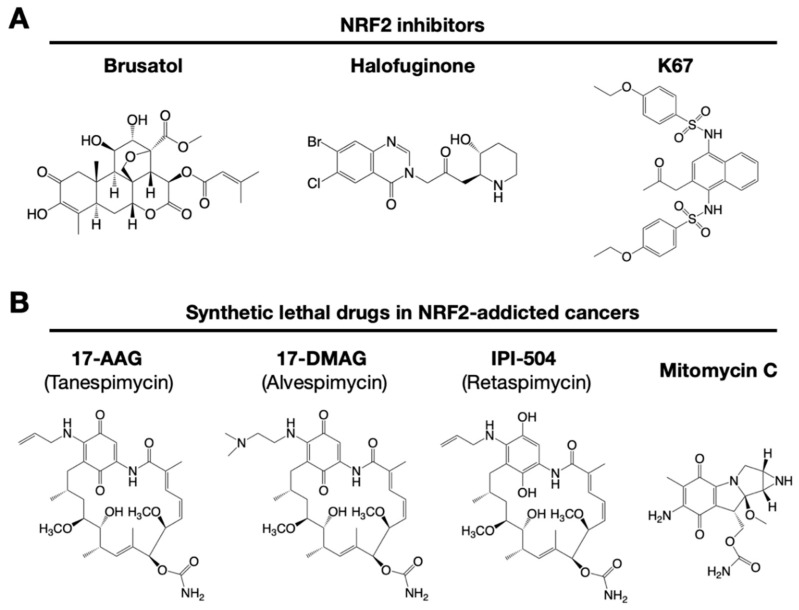
Small chemical inhibitors targeting NRF2. (**A**) NRF2 inhibitors: brusatol, halofuginone and K67. (**B**) Synthetic lethal drugs for NRF2-addicted cancers: 17-AAG (tanespimycin), 17-DMAG (alvespimycin), IPI-504 (retaspimycin) and mitomycin C.

Similarly, NOTCH3 (neurogenic locus notch homolog protein 3) was recently found to play an important role in carcinogenesis under the regulation of NRF2 [79]. NRF2 directly upregulates *NOTCH3* mRNA expression, and both NRF2- and NOTCH3-positive cancers show poor prognosis. These observations indicate that there are multiple pathways to consider when developing NRF2 inhibitors. A precise and deep understanding of the molecular mechanisms that regulate NRF2 activation is critical for the development of drugs targeting NRF2-addicted cancers. Some representative small molecule NRF2 inhibitors are shown in Figure 4.

### 6.2. Small Molecular Weight NRF2 Inhibitors

It has been reported that brusatol enhances the efficacy of chemotherapy by inhibiting NRF2 [80] (Figure 4A). Brusatol provokes the rapid and transient inhibition of NRF2 through a KEAP1-independent posttranscriptional mechanism [81]. The mechanism appears to be not through direct NRF2 inhibition but through inhibition of protein translation [82]. This action of brusatol overcomes chemoresistance in cancer cells. By inhibiting NRF2, brusatol sensitizes cells to chemical stress provoked by 2,4-dinitrochlorobenzene (DNCB), iodoacetamide (IAA), and *N*-acetyl-*p*-benzoquinone imine (NAPQI), the hepatotoxic metabolite of acetaminophen. Whereas brusatol serves as a valuable experimental tool for inhibiting NRF2, the risks presented by its therapeutic use need to be considered, especially its potential for enhancing the sensitivity of nontargeted cells.

Using high-throughput screening of a chemical library, febrifugine was found to inhibit NRF2 activity [83]. Halofuginone is a racemic halogenated febrifugine derivative that was artificially synthesized as a less toxic compound [84]. Halofuginone represses global protein synthesis via the amino acid starvation response elicited by the inhibition of prolyl-tRNA synthetase. As NRF2 is a very short-lived protein even in NRF2-addicted cancer cells, blocking general protein synthesis halts NRF2 accumulation. As an NRF2 inhibitor, halofuginone enhances the chemosensitivity of cancer cells by suppressing NRF2 accumulation.

### 6.3. K67: Disrupting the KEAP1 and P62 Interaction

Accumulated P62 and LC3-II (microtubule-associated protein 1A/1B-light chain 3) are typical markers of impaired autophagy. Phosphorylated P62 accumulates in hepatitis C virus-positive hepatocellular carcinoma (HCC) [85]. In hepatitis and cirrhosis, which are pre-HCC diseases, simultaneous accumulation of P62 and KEAP1 does not frequently occur [86]. However, both P62- and KEAP1-positive lesions are detected in approximately 25% of human HCC and adjacent tissues. The P62 expression level is positively correlated with high levels of NRF2 and NQO1 expression in cultured human HCC lines.

Liver-specific *Pten*-knockout mice (Pten^flox/flox^: Albumin-Cre; Pten-Alb mice) are liver disease models used to develop steatosis, nonalcoholic steatohepatitis (NASH) and liver cancers stepwise [87]. Showing very good agreement with the mouse model, human NASH shows a decreased expression of *PTEN* mRNA compared to that in normal human liver [88]. In Pten-Alb mice, p62 accumulation elevates the NRF2 level, at least partially, and NRF2 target genes are upregulated [78]. These results imply that, in human cases, accumulated P62 may be a therapeutic target in *PTEN*-decreased NASH and HCC.

K67 (2-acetonyl-1,4-bis[(4-ethoxybenzenesulfonyl)amino]naphthalene) is an analog of compound 16 (Cpd16; see details in Section 7.2) (Figure 4A). K67 is an NRF2 inhibitor that disturbs the PPI formed by KEAP1 and P62 phosphorylated at Ser349 in humans (S351 in mice) [89]. K67 effectively inhibits cellular proliferation in HCC, expressing highly phosphorylated P62. Moreover, novel K67 derivatives inhibited the interaction of KEAP1 and phosphorylated P62 [90]. These derivatives increase the sensitivity of cancer cells to anticancer drugs, such as the tyrosine kinase inhibitors sorafenib or regorafenib. These results suggest that K67 derivatives have the potential to be chemosensitizers by inhibiting NRF2 and the expression of NRF2 target genes.

### 6.4. Chemicals That Show Synthetic Lethality with NRF2

NRF2-addicted cancers show constitutively upregulation of NRF2 target genes. A cell culture system was established in which KEAP1-deleted cells and KEAP1-expressing normal cells were cocultured, and their proliferation was monitored by the distinct colors of fluorescence [91]. Drug screenings that aim to identify synthetic lethal chemical compounds that specifically kill cancer cells with intrinsically high NRF2 activity have been identified. Three geldanamycin-derived heat shock protein 90 (HSP90) inhibitors are synthetically lethal to NRF2-expressing cells (Figure 4B): 17-AAG (17-N-allylamino-17-demethoxygeldanamycin; tanespimycin), 17-DMAG (17-dimethylaminoethylamino-17-demethoxygeldanamycin; alvespimycin), and IPI-504 (the hydroquinone form of 17-AAG, retaspimycin). These two benzoquinone-containing compounds, i.e., 17-AAG and 17-DMAG, are converted into hydroquinones in NRF2-addicted cancer cells in which the expression of drug-metabolizing enzymes is specifically upregulated.

In a similar screening, mitomycin C was found to be a synthetic lethal compound in cells with high NRF2 activity [74]. Quite intriguingly, 17-AAG and mitomycin C exert synergistic effects. Thus, geldanamycin-based compounds and mitomycin C are candidates for drug repositioning to target currently undruggable NRF2-addicted cancers. The use of these drugs in NRF2-addicted cancers may avoid unexpected side effects to normal cells to the greatest extent possible.

## 7. NRF2 Inducers for Cancer Chemoprevention and Cancer Treatment

### 7.1. Electrophilic and Oxidative Cysteine Modifiers

**Figure 5:**
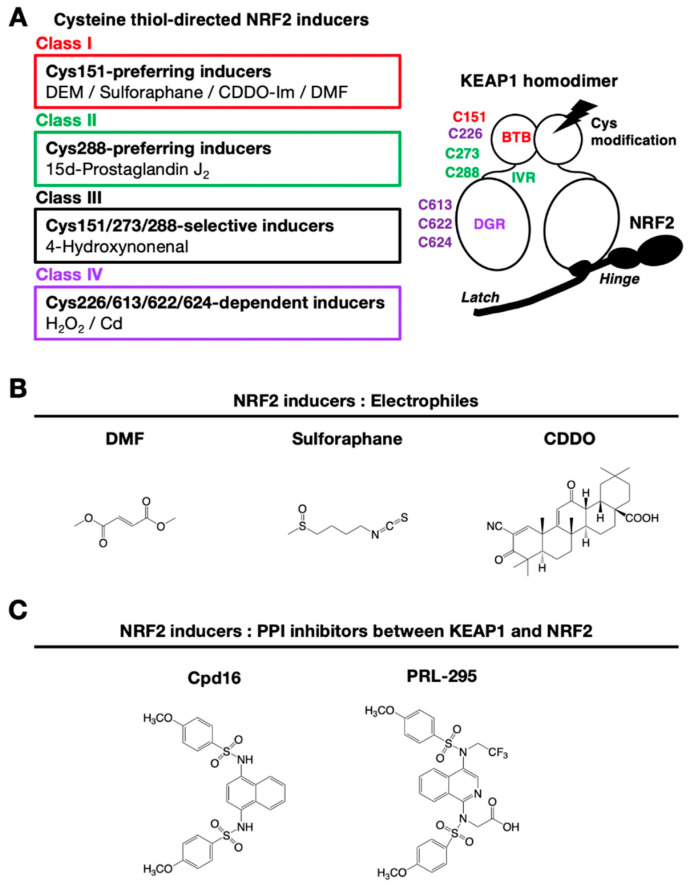
Multiple types of chemical inducers for NRF2. (**A**) Classification of cysteine thiol-directed NRF2-inducers. (**B**) Electrophilic NRF2-inducers: DMF, sulforaphane and CDDO. (**C**) NRF2-inducers that inhibit the protein–protein interaction of KEAP1 and NRF2: compound 16 (Cpd16) and the derivative, PRL-295.

KEAP1 is a multiple cysteine-based sensor protein that detects electrophilic and oxidative stresses. Electrophilic NRF2-inducers lead to NRF2 activation through cysteine-thiol modifications, which inactivate the ubiquitin E3 ligase activity of KEAP1. Electrophilic NRF2-inducers have been found to interact with specific cysteine residues of KEAP1 [8]. Electrophilic and oxidative NRF2-inducers are classified into four classes based on interacting cysteine residues (Figure 5A). Class I consists of Cys151-preferring inducers such as DEM, DMF, sulforaphane and CDDO-Im, Class II consists of Cys288-preferring inducers such as 15d-prostaglandin J_2_, Class III consists of C151/273/288-selective inducers such as 4-hydroxynonenal, and Class IV consists of C226/613/622/624-dependent inducers.

Chemical structures of representative NRF2-inducers classified in Class I are shown in Figure 5B. In addition to electrophilic inducers, ROS are also found to interact with the reactive cysteine residues. Indeed, elaborate analyses have clarified that hydrogen peroxide (Class IV inducer) interacts with Cys226/613/622/624 in KEAP1 [7]. Cysteine 622 and 624 behave as one unit of cysteine; therefore, there are three cysteine units involved in the hydrogen peroxide sensing by KEAP1. Two of the three cysteine units form a disulfide bond upon exposure to hydrogen peroxide, which then inactivates the ubiquitin ligase activity of KEAP1. This redundant use of cysteine residues during hydrogen peroxide sensing establishes an elaborate fail-safe mechanism [7].

### 7.2. Inhibitors of the PPI Formed by NRF2 and KEAP1

A non-covalent inhibition strategy avoids toxic side effects due to nonspecific cysteine modification by electrophilic NRF2-inducers. The development of new generation NRF2-inducers focuses on the disruption of the PPI formed by KEAP1 and NRF2. Capitalizing on the deep understanding of the molecular mechanism by which KEAP1–NRF2 interacts, the structure-based design of PPI inhibitors is possible. PPI inhibitors are desirable drugs for the treatment of oxidative stress- and inflammatory stress-related diseases.

The first identified KEAP1–NRF2 PPI chemical inhibitor was Cpd16 [*N*,*N’*-1,4-naphthalenediylbis(4-methoxybenzenesulfonamide)], which is a naphthalene-based compound that interacts with the specific pocket within the KEAP1 DGR domain (Figure 5C) [92]. Based on Cpd16, optimization of NRF2-inducers for clinical drugs has been achieved. Improved compounds with naphthalene-based structures have been reported [93]. Replacement of naphthalene with a 1,4-isoquinoline scaffold yields an improved mutagenic profile without reduced potency, stability or solubility [94]. The prototype 1,4-isoquinoline-based compound is hydrophilic and possesses negatively charged carboxylate groups that limit membrane permeability at physiological pH, but replacement of a carboxymethyl group with a fluoroalkyl group increases its metabolic stability, lipophilicity, and cellular activity (PRL-295) [95].

### 7.3. Cancer Treatment with NRF2-Inducers

We surmised that KEAP1 expression levels in the microenvironment might affect tumor progression through NRF2 activation. To verify this notion, we exploited the *Kras*^G12D^-driven lung adenocarcinoma system [96] and examined two mouse models with different NRF2 activity levels in the presence of adenovirus expressing Cre recombinase [97]: *KEAP1*^flox-A^ mice [17] and *KEAP1*^flox-B^ mice [98]. The *KEAP1*^flox-A^ mice exhibit generalized hypomorphic KEAP1 knockdown and therefore show both general NRF2 activation and additional tumor-specific NRF2 activation induced by Cre recombinase, while the *KEAP1*^flox-B^ mice do not have this hypomorphic trait and show only Cre-mediated tumor-specific NRF2 activation.

Importantly, in *KEAP1*^flox-A^: Cre mice, NRF2 activation in bone marrow-derived host immune cells leads to tumor suppression [97]. NRF2-inducers seem to be effective in the microenvironment, and this observation may indicate its therapeutic potential, especially in combination with NRF2 inhibitors or other strong anticancer drugs. There may be a new cancer therapy for NRF2-addicted cancers utilizing both NRF2 inhibitor and inducer. In this protocol, NRF2 is repressed during the anticancer chemotherapy period by the NRF2 inhibitor and then activated by the NRF2-inducer during the resting periods when the strong anticancer agents plus NRF2 inhibitor treatment are ceased. This challenging protocol is still under consideration in basic science. There are hurdles to be cleared from bench to bedside.

In addition to cancer treatment via the activation of NRF2 in microenvironments, there are ample lines of evidence from mouse experiments showing that NRF2-inducers are effective in cancer chemoprevention [99,100]. Indeed, in animal experiments, CDDO-Im substantially protects against aflatoxin B_1_-induced liver cancers [101].

## 8. Self and Others in Cancer Cell Society

### 8.1. Cell Competition and NRF2

In most cases, tissues and organs are composed of several types of cells, and they communicate with each other. Multiple types of cells in tissue form a cell society, and in the process of cancer development, they behave distinctly and uniquely. Therefore, understanding the mechanisms by which cells acquire the ability to be cancer cells, how the initiated cell proliferates and selectively survives in the cell society is critically important to explore strategies to eliminate cancers.

The concept of cell competition was first proposed in 1975, utilizing mutants of *Minute* encoding a ribosomal protein in *Drosophila* [102]. During the mosaic colocalization of wild-type and *Minute*-mutant cells during wing development, the mutant cells were positively eliminated, and ultimately, the wild-type cells constituted normal wings. In contrast, abnormal wings were formed in the presence of only *Minute*-mutant cells. These observations implied that wild-type and mutant cells somehow communicate with each other.

Almost 30 years later, the specific elimination of the *Minute* mutant cells was found to be the result JNK (c-Jun N-terminal kinase)-dependent apoptosis at the interface of different cells [103]. Fifteen years after this discovery, it was shown that overexpression of NRF2 primes cells to loser during the cell competition between wild-type and *Minute*-mutant cells [104]. Cell competition is the process leading to cell elimination in heterogeneous tissues, resulting in the fitness selection of winners and losers, and a new role of NRF2 has emerged, suggesting that the expression levels of NRF2 may determine the fate of cells as winners or losers in a cell society. In the mouse esophageal carcinogenesis model that is induced by a chemical carcinogen 4-nitroquinoline-1-oxide (4NQO), we reported that tumors are derived from NRF2-intact cells in the co-existence of NRF2-deficient cells and normal cells [105].

Cell competition to eliminate mutant cells occurs not only in tissue development but also in cancer development and aging [106]. In the case of cancers, Ras^V12^-transformed cells are eliminated more efficiently than surrounding normal cells from the epithelial monolayer in the small intestine and pancreas [107]. In this regard, the cellular changes at the early and intermediate stages of preneoplasia progression are considered essential adaptations to the surrounding environment.

### 8.2. Resistant Hepatocyte Model as an Alternative Model for NRF2-Addicted Cancer Analysis

**Figure 6:**
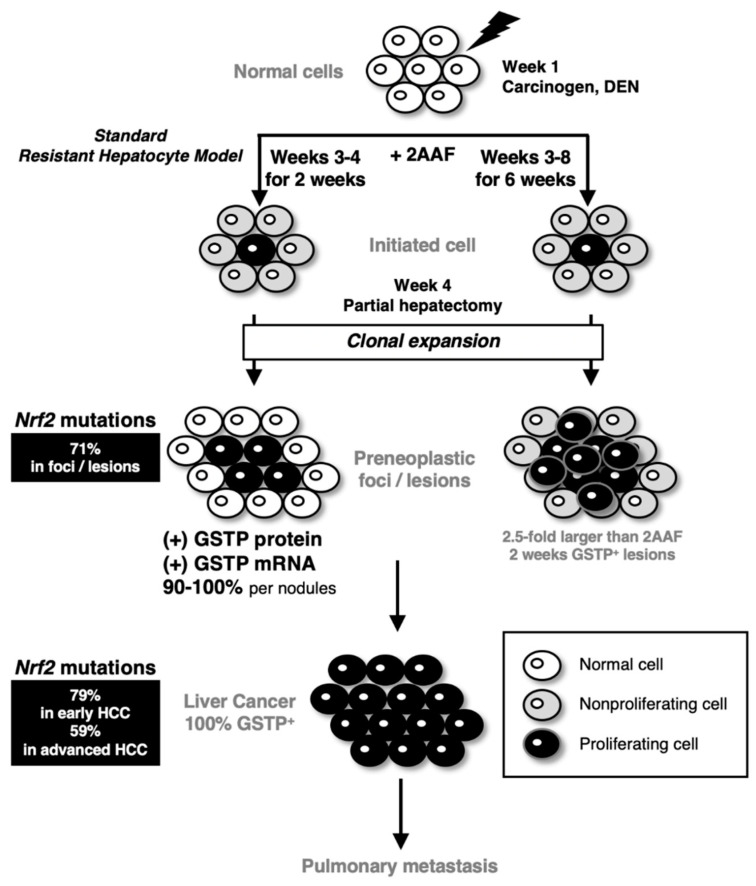
Hepatocarcinogenesis and NRF2 activation in the resistant hepatocyte model.

The rat resistant hepatocyte model, which is also referred to as the Solt–Farber model, was established in 1977 [108,109]. In this model, cancer-initiating cells form glutathione *S*-transferase P (GSTP)-positive preneoplastic foci, and ultimately, these foci develop into HCC (Figure 6). This experimental hepatocarcinogenesis model is generated upon the single administration of diethylnitrosamine (DEN) at week 1, followed by short-term dietary exposure to the mitosis-inhibitory agent 2-acetylaminofluorene (2AAF) at weeks 3–4 (for two weeks) and a 70% partial hepatectomy as a proliferative stimulus at week 4. When proliferation of hepatocytes that surround the foci was inhibited by prolonged 2AAF administration from weeks 3 to 8 (for 6 weeks) [110], the sizes of the GSTP-positive foci/nodules increased approximately 2.5-fold at week 8 compared with those obtained through the standard protocol. This phenomenon suggests the presence of cell competition between the cancer-initiating cells and the surrounding normal hepatocytes in the resistant hepatocyte model (Figure 6).

The resistant hepatocyte model is an experimental hepatocarcinogenesis model consisting of a combination of the carcinogen DEN at week 1, the mitosis-inhibitory agent 2AAF at weeks 3–4 and partial hepatectomy at week 4. In the standard protocol, a DEN-initiated cell is surrounded by cells whose proliferation is inhibited by 2AAF. Partial hepatectomy stimulates proliferation, and the initiated cells undergo clonal expansion to form preneoplastic foci and lesions. Almost all preneoplastic foci are positive for GSTP protein/mRNA. Finally, GSTP-positive hepatocellular carcinoma (HCC) develops in the resistant hepatocyte model. Metastasis to the lung is observed. When 2AAF is continuously exposed from weeks 3 to 8, the preneoplastic foci/lesion sizes become larger approximately 2.5-fold at weeks 8 than those at weeks 3–4 [110]. All foci/lesions harbor GSTP protein and mRNA after the 2AAF exposure. Withdrawal of 2AAF at weeks 5–8 inhibits the tumor-initiated cell expansion. These results support the presence of cell competition in which the growth of surrounding cells suppresses the expansion of tumor-initiated cells. Of note, somatic mutations of the *NRF2* gene are found in very high-frequency in the early preneoplastic foci/lesions and both early and advanced HCCs [111]

Using this resistant hepatocyte model, Columbano and colleagues made a breakthrough, showing that somatic and missense *NRF2* mutations in the DLGex and ETGE motifs are frequently observed in early preneoplastic lesions and early and advanced HCCs [111]. NRF2-addicted HCCs are also secondarily detected as lung metastasis. Constitutive NRF2 activation by somatic mutations in the *NRF2* gene coincides well with the overexpression of NRF2-target gene products, including GSTP, in preneoplastic foci and HCCs. These induced expressions of antioxidant enzymes have been shown as an adaptive response in this resistant hepatocyte model [112]. Whole-exome sequencing has identified 6.4% of the somatic mutations in *NRF2* in HCC patients [113]. In human HCC, *CTNNB1* (*Catenin b1*; coding *β-Catenin*; 32.8%) and *TP53* (20.8%) mutations are more frequent than *NRF2*. In the resistant hepatocyte model, *β-catenin* mutations were found in adenomas and HCCs only at the late-stage. *NRF2* mutation seems to be an early marker in the hepatic carcinogenesis of rats.

Whereas NRF2-addicted cancers have been found in the analyses of non-small cell lung carcinomas and certain other cancers in humans [55], the resistant hepatocyte model is an alternative and important example of NRF2-addicted cancers. We believe that the latter will contribute to the analyses of HCC progression and development.

## 9. Closing Remarks

The KEAP1–NRF2 system has been intensively studied in the context of cancer chemoprevention, which revealed that the system activates antioxidant and detoxicating enzymes and protects our bodies from chemical carcinogens [100]. The discoveries of somatic mutations in the *KEAP1* and *NRF2* genes [55,69] and NRF2-addicted cancers [54] established other lines of important cancer-related studies related to the KEAP1–NRF2 system; therefore, this system has become very popular in cancer science. NRF2 exhibits activity as both an oncogene and a tumor suppressor, depending on the context. NRF2 activation in cancer cells is brought by not only somatic mutations in *NRF2* or *KEAP1* genes but also several unique mechanisms, including oncometabolites, exon skipping, and promoter methylation, in NRF2-addicted cancers.

The NRF2-addicted cancers hijack intrinsic roles that NRF2 plays in cytoprotection, including antioxidative and anti-electrophilic responses as well as metabolic reprogramming. Cancer cells acquire marked advantages upon NRF2 activation and are able to survive under severe and limited microenvironments. Another intriguing finding is that NRF2 activation in host immune cells significantly suppresses cancer cell growth, indicating that NRF2 activation also has the potential to be therapeutic for cancers. Thus, it is now clear that the KEAP1–NRF2 system makes extensive contributions to both cancer development and suppression. These observations support the notion that both NRF2 inhibitors and inducers are useful for the treatment of NRF2-addicted cancers.

## Data Availability

No new data were created or analyzed in this study. Data sharing is not applicable to this article.
